# Risk Management in Drug-Device Combination Product Development

**DOI:** 10.1007/s43441-022-00425-w

**Published:** 2022-06-26

**Authors:** Robin Kumoluyi, Amit Khanolkar

**Affiliations:** Janssen : Pharmaceutical Companies of Johnson & Johnson, 1125 Trenton-Harbourton Road, Titusville, NJ 08560 USA

**Keywords:** Combination products, Integrated risk management, Product experience, Regulatory lifecycle

## Abstract

Breakthrough innovations in drug and biologic based therapies as well as technological developments in design and development of medical devices have led to the rapid rise in the use of combination products in the last few decades. The resulting maturity of combination products as a global market category has led to 3 key trends: expanding adoption of medical devices for drug delivery, increasing complexity, and growing awareness of product experience and risk. Adoption of integrated combination product risk management enables robust product development and manufacturing strategies as well as facilitates alignment with regulators for efficient regulatory pathways and predictable outcomes.

## Introduction

Continuing innovation in drug and biologic based therapies coupled with technological developments in design and development of delivery systems have resulted in a proliferation of drug-device combination products. The past 30 years have seen breakthrough innovation in drug and biologic based therapies which have created opportunities for novel delivery techniques. This, coupled with technological developments in design and development of delivery systems, especially due to advances in material sciences and additive manufacturing, have resulted in a proliferation of drug-device combination products. The development of novel materials like nanoparticles, hydrogels, pH responsive polymers, micelles, and dendrimers have enabled the development of targeted delivery of drugs and biologics. Device components as well as drug dosage forms can be rapidly prototyped, evaluated in clinicals as well as manufactured commercially using additive technologies like 3D Printing, which have led to an increase in combination products being commercialized.

Drug-Device Combination products are a result of convergence of two traditionally independent constituents—drug and medical device. A drug-led combination product is essentially a drug delivery mechanism (device) combined with a drug source that transfers the drug onto or into the patient’s body for diagnostic or therapeutic purposes. Drug delivery devices bring to the table numerous advantages including improving adherence to the drug regimen, provide localized delivery, drive patient centricity and satisfaction, create intellectual property, a way to differentiate the drug product in a competitive market and ultimately leading to innovation that benefits public health. One such example is SPRAVATO® (Esketamine), an antagonist of the N-methyl-D-aspartate (NMDA) glutamate receptor, which is delivered via a nasal spray device [1].

## Combination Product Global Trends

The maturity of drug-device combination products globally has resulted in three key trends-

1. **Expanding Adoption of Medical Devices for Drug Delivery** Medical devices present an attractive option for the delivery of drug products on multiple fronts, whether it is for convenience, privacy, or safety of the patient as well as health care provider. The increasing adoption of medical devices in drug delivery is evident from the number of combination product submissions to the United States Food and Drug Administration (US FDA) which increased from 317 in 2014 to 518 in 2019, which represents a ~ 10% growth year over year in those 5 years [2]. Similarly, the global combination product market value continues to grow at a compound annual growth rate of 7% and estimated to be valued at $139 billion by 2025 [3].
2. **Increasing Complexity of Drug Delivery Systems** The growth of new therapeutics and delivery technologies have also led to novel product/system configurations and the resultant increase in complexity in four areas, namely Technical Development, Product Quality, Regulatory, and Supply Chain.
  - *Technical Development* Integrating the constituent parts of the combination product, namely the drug and the device in complex configurations, for example an electro-mechanical patch pump with embedded software for the delivery of highly viscous biologics, requires unique considerations in characterization of the product constituents, their interactions, and the combination product system level performance.
  - *Product Quality* Establishing drug and device performance requirements during clinical and commercial stages and ensuring that the drug and device performance is maintained throughout the life of the product requires defining and adopting appropriate quality systems for the constituent parts and the combination product. For example, the development of an autoinjector for the delivery of a biologic, a single entity combination product, requires the adoption of quality systems for the biologic constituent as well as application of design controls and purchasing controls for the development of the autoinjector. Additional quality system elements, such as CAPA and Management Responsibility under 21 CFR 820 likewise need to be established.
  - *Regulatory* Identifying the most efficient and streamlined regulatory pathway for complex product configuration depends on several factors including its primary mode of action, regulatory precedents, and market experience with current products.
  - *Supply Chain* Complexity in product configurations requires employing complex global supply chain and logistics. Combination product supply chain typically involve manufacturing of the drug substance, the excipients, the drug product, the device constituents, the assembly of the constituents and subsequent packaging and labeling at various sites in a global supply chain. Additionally different product configurations, regulatory status, and labeling differences by country requires additional considerations for shipping, warehousing, distribution etc.
3. **Growing Awareness of Product Experience and Risk** Over the years, health authorities around the world have required and adopted transparency measures leading to increasing availability and access to market performance data of healthcare products in the public domain. The US FDA’s Adverse Event Reporting System (FAERS) database is a web-based tool containing detailed listing of Adverse Events in a dashboard format. Open-source pharmacovigilance data analysis platforms like Open Vigil have enhanced public access to global health authority databases. With the exponential rise in public participation on social media, especially social networking sites, micro-blogs, wikis, and media sharing sites, healthcare product experience and risks are routinely discussed on platforms with global reach.

## Combination Product Risk Management

**Fig. 1:**
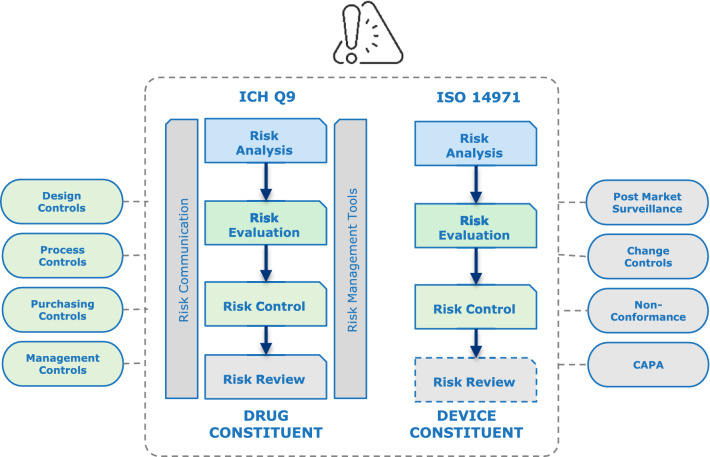
Simplified view of integrated risk management process during combination product lifecycle

Risk management is an ongoing process throughout the product lifecycle which includes proactive identification of hazards and harms, evaluation of those risks, mitigating and controlling the risks, conducting the risk benefit analysis, and continually reviewing the adequacy and effectiveness of the risk control measures as well as any new risks. For drug-device combination products, the integrated risk management process involves elements of drug and device risk management following Quality Risk Management (ICH Q9) and Medical Device Risk Management (ISO 14971), respectively. Combination Products Risk Management (AAMI TIR105:2020) [4] and Medical devices-Guidance on the application of ISO 14971 (ISO TR 24971:2020) [5] are helpful references on the integrated approach for drug-device combination products. The relationship between risk management and other development and lifecycle management processes are shown in Fig. 1. The integrated risk management framework is embedded within design controls, manufacturing process controls, purchasing controls, and management control processes. The risk management files are updated in the lifecycle as new information becomes available from post-market surveillance, change control and corrective and preventive action (CAPA) processes.

The key differentiating aspect of drug-device combination product risk management is the evaluation and mitigation of risks arising from the interaction of the drug and device constituents and the combination product, in addition to risks from the constituent parts.

Product risks that manifest in their commercial use typically originate upstream in product design or manufacturing. System level failure usually occurs through a series of events involving use error and/or latent conditions which can be traced to design or manufacturing [6]. The use of integrated risk management in upstream design and manufacturing ensures the identification, assessment, and control of risks when flexibility for change implementation is maximum.

The application of risk management in design and development enables the identification and control of critical requirements for safety and efficacy. For example, design and process hazards can be identified using Failure Modes and Effects Analysis (FMEA), in support of Harms/Hazard Analysis. The insights from such assessments serve as inputs into the development of design of experiments and product characterization studies, and ultimately control strategies. Similarly, formative, and summative human factor studies are designed based on a Use-Related Risk Analysis.

## Combination Product Regulatory Lifecycle

The regulatory designation of a combination product, as a drug, device, or both, has a significant impact on the quality systems adopted as well as the product development and commercial go-to-market strategies. If the combination product is regulated as a device, then its risk classification defines its regulatory and quality strategy.

The increasing complexity of drug delivery systems, discussed above, results in increasing regulatory ambiguities. Navigating this ambiguity is possible by adopting a robust regulatory strategy based on risk-informed scientific practices, especially in the application of performance standards to the combination product. Collective industry experience indicates proactive alignment, transparency, and open engagement with regulators on risk-based approaches to product development, pre-market submissions and lifecycle management results in predictability of outcomes.

The US FDA, for example, has established several pathways for expedited regulatory review and approval of combination products based on both the drug and device primary modes of action (PMOA). For combination products with drug PMOA, these regulatory pathways include the *Fast-Track Designation, Breakthrough Therapy Designation, Accelerated Approval,* and *Priority Review Designation* [7]*.* Similarly for combination products with device PMOA, the regulatory pathways include the *Breakthrough Device Program* [8] and the *Safer Technologies Program* [9]*.*

As more and more novel therapies and drug delivery systems are developed to address unmet patient needs, thorough yet streamlined reviews and approval of these products is critical in the interest of public health as well as efficient utilization of resources at regulatory bodies which are funded by tax dollars.

## Conclusion

The exponential rise in the development, approval, and use of combination products has been fueled by breakthrough innovations in drug and biologic based therapies as well as technological developments in design and development of delivery systems. The maturity of combination products as a market category has led to 3 key global trends: expanding adoption of medical devices for drug delivery, increasing complexity and growing awareness of product experience and risk. Integrated product risk management enables robust product development and manufacturing strategies as well as efficient regulatory pathways.
